# DISSECTING THE HETEROGENEITY OF SENESCENCE: PROFILING PRIMARY AND SECONDARY SENESCENT STATES USING SINGLE-CELL AND SPATIAL TRANSCRIPTOMICS

**DOI:** 10.1093/geroni/igae098.1145

**Published:** 2024-12-31

**Authors:** Nicola Neretti

**Affiliations:** Brown University, Providence, Rhode Island, United States

## Abstract

Cellular senescence is a state of irreversible cell cycle arrest, contributing to age-associated tissue function decline due to the accumulation of senescent cells. The Senescence Associated Secretory Phenotype (SASP), a collection of signaling molecules and growth factors secreted by these cells, plays a significant role in the sterile inflammation seen in aging tissues. Recent studies, both in vivo and in vitro, have demonstrated that senescence is a heterogeneous phenotype, varying based on cell type, induction method, and duration of senescence. An important aspect of this heterogeneity is the distinction between primary and secondary senescence; primary senescent cells can induce nearby cells into a secondary senescent state, which shares some markers with primary senescence but differs significantly in others, such as the SASP. In this study, we explore the evolution of primary and secondary senescence over time by profiling individual cells during the early stages of senescence establishment using single-cell transcriptomics. We have developed an AI/ML methodology capable of classifying cells into primary and various forms of secondary senescence. This methodology is compared with existing methods like SenMayo and SenSig, and applied to time-course scRNA-seq data to determine the proportion of cells in different senescent states. Additionally, we present findings on the identification of senescent cells in vivo using spatial transcriptomics to study the aging mouse liver.

